# Supplementary material for: Integrated fire management as an adaptation and mitigation strategy to altered fire regimes
Source: Commun Earth Environ. 2025 Mar 15;6(1):202. doi: 10.1038/s43247-025-02165-9 (PMC11910340; doi:10.1038/s43247-025-02165-9)
Supplement: Supplementary file 2 — Supplementary Information [file 43247_2025_2165_MOESM2_ESM.pdf]

Supplementary table S1. Examples of local to regional initiatives of IFM (represented in Figure 1).

Continent	Country	Region	Latitude	Longitude	IFM Objectives	References
Africa	Namibia	East Caprivi	21.6696	18.116	LL, LR, W	[1,2]
Africa	South Africa	Kruger National Park	-23.988	31.555	E, LR, W	[3]
Africa	Zambia	Western Province, Kafue National Park	-14.499	26.168	LL, LR, W	[1,4]
Asia	China	Wenyime in Dayao county, Chuxiong Yi Autonomous Prefecture	25.895	100.977	LL, LR, W	[5]
Asia	India	Mizoram, NE India	23.479	93.320	LL, LR	[6]
Asia	Japan	Kaida Highland	35.954	137.654	E, LL, LR, W	[7]
Asia	Japan	Mt. Sanbe	35.128	132.616	E, LL, LR, W	[8]
Asia	Philippines	Mountain Province in the Cordillera of Luzón	17.190	121.444	LL, W	[1,9]
Asia	Indonesia	Riau, Sumatra (Jambi and Palembang) and Central Kalimantan	0.29335	101.707	E, LL, LR, W	[10–12]
Central America	Mexico	Chiapas, Sepultura Reserve; Montebello National Park	16.175	-93.622	E, LL, W	[13,14]
Central America	Mexico	Mexico City (Ajusco Project), Puebla	19.244	-99.254	E, LL, W	[15,16]
Europe	France	Pyrenees-Orientales	42.726	2.307	E, LL, W	[17,18]
Europe	Italy	Friuli-Venezia Giulia	46.555	13.306	E, LL, W	[19]
Europe	Italy	Sardinia	40.533	9.311	LL, W	[20]
Europe	Italy	Tuscany	43.724	11.614	E, LL,W	[21]

Europe	Portugal	Meseta Ibérica	41.409	-12.205	LL,LR,W	18
Europe	Portugal	Northern region	41.650	-8.053	LL,LR,W	22
Europe	Portugal	Alto Minho	41.840	-8.559	LL,LR,W	23
Europe	Spain	Catalonia (FireFlocks; GRAF)	42.161	2.675	LL,LR,W	24
Europe	Spain	Andalucia	37.444	-4.899	LL,LR,W	25
Europe	Sweden	Swedish Taiga	60.273	16.900	E,W	26
Europe	Spain	Catalonia (Val d'Aran)	42,72	0,84	E, LL, LR, W	27
North America	Canada	Southern Alberta, SK	50.601	-132.230	E, LL, W	28,29
North America	Canada	Southern BC	51.514	-125.140	E,LR, W	30,31
North America	Canada	Central BC	49.712	-117.720	E, LR, W	32
North America	United States of America	Northern California	41.000	-125.740	E, LR, W	33,34
North America	United States of America	Great Plains	31.689	-107.090	E, LL, LR, W	35,36
North America	United States of America	Florida/Southeast	31.249	-92.544	E, LR, W	37,38

Oceania	Australia	Queensland/NT	-13.117	141.878	E, LL, C, W	39
Oceania	Australia	Western Desert	-23.102	120.889	E, LL, LR, W	33
Oceania	Australia	NSW/Victoria	-34.260	150.447	E, LL, LR, W	40
South America	Bolivia	Chiquitania	-17.757	-63.635	E, LL, LR, W	41
South America	Bolivia	Lomerio	-16.826	-61.859	E, LL, LR, W	42
South America	Brazil	Roraima	-6.283	43.188	E, LL, LR, W	43
South America	Brazil	Cerrado	-8.397	-47.405	E, LL, LR, W	44
South America	Venezuela	Gran Sabana, Canaima National Park, North Amazonia	5.500	-61.500	E, LL, LR, W	43,45

References

1. Goldammer, J. G. *et al.* Community participation in integrated forest fire management: experiences from Africa, Asia and Europe.
2. Humphrey, G. J., Gillson, L. & Ziervogel, G. How changing fire management policies affect fire seasonality and livelihoods. *Ambio* **50**, 475–491 (2021).
3. Marais, C., Le Maitre, D. and Frost, P., 2015. The Working on Fire Programme: Mainstreaming integrated veld and forest fire management into economic development. In XIV World Forestry Congress (pp. 7-11).

4. The Nature Conservancy. Fighting Fire with Fire in Zambia. *The Nature Conservancy* https://www.nature.org/en-us/about-us/where-we-work/africa/stories-in-africa/fighting-fire-with-fire-in-zambia/ (2018).
5. Lichang, Z., Long, W., Yaqiao, Z. & Caizhen, L. Community-Based Forest Fire Management in Wenyime Village, Sanchahe Township, Dayao County, Chuxiong Yi Autonomous Prefecture, Yunnan Province, China. *Case Stud.* (2001).
6. Darlong. Traditional community-based fire management among the Mizo shifting cultivators of Mizoram in northeast India. *Communities Flames Proc. Int. Conf. Community Involv. Fire Manag.* (2002).
7. Nagata, Y. K. & Ushimaru, A. Traditional burning and mowing practices support high grassland plant diversity by providing intermediate levels of vegetation height and soil pH. *Appl. Veg. Sci.* **19**, 567–577 (2016).
8. Sanbe Area Tourism Promotion Association. Nishinohara Field Burning | Mt. Sanbe Area Tourist Information - A Sacred Mountain in the Daisen-Oki National Park. https://www.sanbesan.jp/en/touristspot/4668/.
9. Ancog, R. C., Florece, L. M. & Nicopior, O. B. Fire occurrence and fire mitigation strategies in a grassland reforestation area in the Philippines. *For. Policy Econ.* **64**, 35–45 (2016).
10. Nurhidayah, L., Astuti, R., Hidayat, H. & Siburian, R. Community-Based Fire Management and Peatland Restoration in Indonesia. in *Environmental Governance in Indonesia* (eds. Triyanti, A., Indrawan, M., Nurhidayah, L. & Marfai, M. A.) 135–150 (Springer International Publishing, Cham, 2023). doi:10.1007/978-3-031-15904-6_8.
11. Purnomo, H. *et al.* Community-based fire prevention and peatland restoration in Indonesia: A participatory action research approach. *Environ. Dev.* **50**, 100971 (2024).

12. Fernandes, A. A. R., Panjaitan, R. B. & Solimun. The effect of community and company participation and implementation of good forest fire governance on the forest fire policy in Indonesia. *J. Sci. Technol. Policy Manag.* **10**, 102–115 (2018).
13. Guevara-Hernández, F. *et al.* Traditional fire use, governance and social dynamics in a Biosphere Reserve of Chiapas, Mexico. *La Pensée* **75**, (2013).
14. Ponce-Calderón, L. P. *et al.* Fire management in pyrobiocultural landscapes, Chiapas, Mexico. *Trop. For. Issues* 53–59 (2022) doi:10.55515/ABWJ7126.
15. Rodríguez-Trejo, D. PAST, PRESENT AND NEXT STEPS OF THE AJUSCO PROJECT: FIRE ECOLOGY, RESTORATION AND INTEGRAL FIRE MANAGEMENT IN CENTRAL MEXICO.
16. Trejo, D. A. R. & Reyes, A. C. Advances in integrated fire management in Central Mexico. in *Proceedings of the fourth international symposium on fire economics, planning, and policy: Climate change and wildfires Pacific southwest* 338–356 (Citeseer, 2013).
17. Fernandes, P. M. *et al.* Prescribed burning in southern Europe: developing fire management in a dynamic landscape. *Front. Ecol. Environ.* **11**, e4–e14 (2013).
18. *Best Practices of Fire Use: Prescribed Burning and Suppression Fire Programmes in Selected Case-Study Regions in Europe*. (European Forest Institute, Joensuu, 2010).
19. Ascoli, D. *et al.* Fire-smart solutions for sustainable wildfire risk prevention: Bottom-up initiatives meet top-down policies under EU green deal. *Int. J. Disaster Risk Reduct.* **92**, 103715 (2023).
20. Ascoli, D. *et al.* Esperienze di fuoco prescritto in Italia: un approccio integrato per la prevenzione degli incendi boschivi. *For. - J. Silvic. For. Ecol.* **9**, 20 (2012).

21. Life Granatha - Growing avian in Apennine's Tuscany Heathlands | Miglioramento dello stato di conservazione degli uccelli presenti negli habitat di brughiera in Appennino. https://www.lifegranatha.eu/.
22. ICNF - Instituto da Conservação da Natureza e das Florestas. https://www3.icnf.pt/imprensa/fogocontrolado.
23. Oliveira, E. & Fernandes, P. M. Pastoral Burning and Its Contribution to the Fire Regime of Alto Minho, Portugal. *Fire* **6**, 210 (2023).
24. Nuss-Girona, S. *et al.* Fire Flocks: Participating Farmers' Perceptions after Five Years of Development. *Land* **11**, 1718 (2022).
25. Mena, Y., Ruiz-Mirazo, J., Ruiz, F. A. & Castel, J. M. Characterization and typification of small ruminant farms providing fuelbreak grazing services for wildfire prevention in Andalusia (Spain). *Sci. Total Environ.* **544**, 211–219 (2016).
26. Swedish Environmental Protection Agency & EU Commission Life+ Nature. Controlled burning in woodlands - LifeTaiga. https://lifetaiga.se/controlled-burning-in-woodlands/.
27. Oliveres, J., Castellnou, M., Castellarnau, X., Brotons, L., & Duane, A. Time for Managed Wildfire in Europe: The pioneering integrated management plan of the Aran, Pyrenees. Preprint at https://doi.org/10.21203/rs.3.rs-4626315/v1 (2024).
28. Christianson, A., McGee, T. K. & L'Hirondelle, L. How historic and current wildfire experiences in an Aboriginal community influence mitigation preferences. *Int. J. Wildland Fire* **22**, 527–536 (2012).
29. Taylor, S. W., Stennes, B., Wang, S. & Taudin-Chabot, P. Integrating Canadian wildland fire management policy and institutions: Sustaining natural resources, communities and ecosystems. *Can. Wildland Fire Strategy Backgr. Synth. Anal. Perspect.* 3–25 (2006).
30. Nikolakis, W., Welham, C. & Greene, G. Diffusion of indigenous fire management and carbon-credit programs: Opportunities and challenges for "scaling-up" to temperate ecosystems. *Front. For. Glob. Change* **5**, (2022).
31. Cultural Burning And Prescribed Fire. Case studies. *Cultural Burning & Prescribed Fire* https://prescribedfire.ca/case-studies/ (2024).

32. Gathering Voices Society. Gathering Voices Society. *Gathering Voices Society* https://www.gatheringvoices.com.

33. Burr, J. L. Burning across boundaries: comparing effective strategies for collaboration between fire management agencies and Indigenous communities. *Occas. Interdiscip. Stud. Humanit.* **5**, 1–16 (2013).

34. Boisramé, G. F. S., Thompson, S. E., Tague, C. (Naomi) & Stephens, S. L. Restoring a Natural Fire Regime Alters the Water Balance of a Sierra Nevada Catchment. *Water Resour. Res.* **55**, 5751–5769 (2019).

35. Kansas Forest Service. Prescribed Burn Associations. https://kstate.maps.arcgis.com/apps/webappviewer/index.html?id=3eacaaf1a3514d3da2e5215b5dd55f9b.

36. Twidwell, D. *et al.* The rising Great Plains fire campaign: citizens' response to woody plant encroachment. *Front. Ecol. Environ.* **11**, e64–e71 (2013).

37. Crate, S. 2021 ALRI Accomplishment Report.

38. Diaz, J., Fawcett, J. E. & Weir, J. R. The Value of Forming a Prescribed Burn Association (PBA). (2016).

39. Department of Environment and Natural Resources, Australian Northern Territory Government. Aboriginal Carbon Industry Strategy.

40. Firesticks. Firesticks, Cultural burning: healthy communities, healthy landscapes. *Firesticks* https://www.firesticks.org.au/.

41. Ibarnegaray, V., Carlos Pinto, & Natalia Calderón. Community-based fire management in Bolivia: integrating people, knowledge and good practices. *Trop. For. Issues* **61**, (2022).

42. Rodríguez, I., Inturias, M., Masay, E. & Peña, A. Decolonizing wildfire risk management: indigenous responses to fire criminalization policies and increasingly flammable forest landscapes in Lomerío, Bolivia. *Environ. Sci. Policy* **147**, 103–115 (2023).

43. Bilbao, B., Mistry, J., Millán, A. & Berardi, A. Sharing Multiple Perspectives on Burning: Towards a Participatory and Intercultural Fire Management Policy in Venezuela, Brazil, and Guyana. *Fire* **2**, 39 (2019).

44. Mistry, J. *et al.* Indigenous Fire Management in the cerrado of Brazil: The Case of the Krahô of Tocantíns. *Hum. Ecol.* **33**, 365–386 (2005).

45. Bilbao, B. *et al.* An intercultural vision for integrated fire management in Venezuela. *Trop. For. Issues* **61**, (2022).
